# Identification of novel genomic imbalances in Saudi patients with congenital heart disease

**DOI:** 10.1186/s13039-018-0356-6

**Published:** 2018-01-25

**Authors:** Zuhair N. Al-Hassnan, Waad Albawardi, Faten Almutairi, Rawan AlMass, Albandary AlBakheet, Osama M. Mustafa, Laila AlQuait, Zarghuna M. A. Shinwari, Salma Wakil, Mustafa A. Salih, Majid Al-Fayyadh, Saeed M. Hassan, Mansour Aljoufan, Osima Al-Nakhli, Brynn Levy, Balsam AlMaarik, Hana A. Al-Hakami, Maysoon Alsagob, Dilek Colak, Namik Kaya

**Affiliations:** 1 Department of Medical Genetics, King Faisal Specialist Hospital and Research Centre, Riyadh, Kingdom of Saudi Arabia; 2 Department of Genetics, King Faisal Specialist Hospital and Research Centre, MBC: 03, Riyadh, 11211 Kingdom of Saudi Arabia; 3 Division of Pediatric Neurology, Department of Pediatrics, College of Medicine, King Saud University, Riyadh, Kingdom of Saudi Arabia; 4 Heart Center, King Faisal Specialist Hospital and Research Centre, Riyadh, Kingdom of Saudi Arabia; 5 Department of Pathology and Cell Biology, Columbia University, New York, NY USA; 6 Department of Biostatistics, Epidemiology and Scientific Computing, King Faisal Specialist Hospital and Research Centre, Riyadh, Kingdom of Saudi Arabia; 7 College of Medicine, Alfaisal University, Riyadh, Saudi Arabia

**Keywords:** Congenital heart disease, Cervical ankylosis, Hypoplastic thumb, Osteopenia, Fused central vertebrae

## Abstract

**Background:**

Quick genetic diagnosis of a patient with congenital heart disease (CHD) is quite important for proper health care and management. Copy number variations (CNV), chromosomal imbalances and rearrangements have been frequently associated with CHD. Previously, due to limitations of microscope based standard karyotyping techniques copious CNVs and submicroscopic imbalances could not be detected in numerous CHD patients. The aim of our study is to identify cytogenetic abnormalities among the selected CHD cases (*n* = 17) of the cohort using high density oligo arrays.

**Results:**

Our screening study indicated that six patients (~35%) have various cytogenetic abnormalities. Among the patients, only patient 2 had a duplication whereas the rest carried various deletions. The patients 1, 4 and 6 have only single large deletions throughout their genome; a 3.2 Mb deletion on chromosome 7, a 3.35 Mb deletion on chromosome 3, and a 2.78 Mb a deletion on chromosome 2, respectively. Patients 3 and 5 have two deletions on different chromosomes. Patient 3 has deletions on chromosome 2 (2q24.1; 249 kb) and 16 (16q22.2; 1.8 Mb). Patient 4 has a 3.35 Mb an interstitial deletion on chromosome 3 (3q13.2q13.31).

Based on our search on the latest available literature, our study is the first inclusive array CGH evaluation on Saudi cohort of CHD patients.

**Conclusions:**

This study emphasizes the importance of the arrays in genetic diagnosis of CHD. Based on our results the high resolution arrays should be utilized as first-tier diagnostic tool in clinical care as suggested before by others. Moreover, previously evaluated negative CHD cases (based on standard karyotyping methods) should be re-examined by microarray based cytogenetic methods.

## Background

Congenital heart disease (CHD) is the most common anomaly affecting newborns and also leading cause of mortality and morbidity among neonates [1–4]. This group of disorders is predicted to have an incidence rate of 8–9 in every 1000 live birth [4, 5] and leads to ~10% of spontaneous miscarriages [5]. Despite the still largely ambiguous pathophysiology of CHD, genetic factors were found to contribute to the etiology in many cases. In addition, numerous incidents of CHD were found to have chromosomal abnormalities; particularly among cases with associated multiple organ malformations, developmental delays, and growth abnormalities [6, 7]. Interestingly enough such cases are prone to harbor morbidities of additional chromosomal syndromes such as Williams-Beuren and DiGeorge or even monogenic hereditary disorders such as Noonan [6].

Advances in molecular and cytogenetic techniques in the recent years gave rise to tools of higher sensitivity such as single nucleotide polymorphism (SNP) based microarrays [8], array comparative genomic hybridization (aCGH) platforms [9–11] and nextgen sequencing [12] techniques, which are enabling the detection of chromosomal aberrations and sub-microscopic copy number variations (CNVs) on an unprecedented resolution that was not possible with standard and high-resolution karyotyping techniques. This facilitated the discovery of novel pathogenic copy number variations, genes and mutations, and the establishment of genotype-phenotype correlations for various diseases [10, 13–15] including heart defects [16]. The dense coverage of the microarray probes can also be quite helpful in refining breakpoints of novel genomic imbalances as well as further characterization and fine mapping of already known gains and losses in different human chromosomes [15, 17].

It has been well-established that standard microscope based chromosome analysis misses quite many gains and losses due to its low resolution. Hence, aCGH and/or similar array platforms have been proposed to be utilized as a first-tier diagnostic tool for various disorders including autism, intellectual disability and more recently for newborn screenings of CHD patients [18–21]. In this study we screened Saudi CHD patients using high density oligo arrays to identify likely chromosomal imbalances.

## Methods

### Patients

We ascertained 223 patients inflicted with one or more of the following clinical problems: autism spectrum disorder, intellectual disability, heart defects, developmental delay, language delay, and dysmorphic features of unknown origin evaluated at the Kind Faisal Specialist Hospital and Research Center using the institutionally approved IRB protocols (RAC# 2040042, 2,030,046, 2,120,022, 2,080,032). Before the sample collection, the patients and parents were signed the written informed consents. All the patients were clinically examined and underwent a consistent study protocol for with perinatal history, and neurological assessment. The patients also underwent aCGH testing as a first-tier approach and then tested with one of the followings; FISH, standard cytogenetics, and targeted sequencing.

### DNA isolation

Blood samples were collected from all participants. DNA was isolated using PureGene DNA Purification Kit (Gentra Systems, Inc. Minneapolis, MN, US).

### Affymetrix microarrays and analysis

Affymetrix’s Cytogenetics Whole-Genome 2.7 M arrays (Affymetrix Inc., Santa Clara, CA, US) and CytoScan HD arrays were used in the study. Both assays have over 2 million probes that interrogate polymorphic and nonpolymorphic genomic sequences. The assay preparation, scanning, image processing, genotyping, and preliminary data analysis were all done according to manufacturer’s protocols and guidelines. CNV detection was done using Affymetrix’s in-house developed software called “Chromosome Analysis Software” otherwise known as ChAS using the software’s default detection settings for high resolution. Previously reported benign CNVs were excluded from the analysis.

### Cytogenetic banding analysis

The microscope based standard karyotype analysis was performed onTrypsin-Wright (GTW) banded metaphase spreads (at least 20 metaphases were analyzed and 2 were karyotyped using cultured peripheral blood lymphocytes according to standard protocols. Karyotypes were interpreted according to the International System for Human Cytogenetic Nomenclature.

### Array CGH

A custom designed oligonucleotide microarray assay from Agilent (Agilent Technologies, Santa Clara, CA, USA) was utilized for CNV assessment [22]. The assay was developed and tested through an academic laboratory consortium [22]. Human male or female DNA (Promega Corp., Nepean, Canada) was used as a reference control. After DNA GC check, good quality DNA was digested, labelled, and then hybridized onto the custom arrays. Then, the slides were washed and scanned with either Agilent DNA Microarray Scanner (Agilent Technologies) or GenePix 4000B (Molecular Devices, Sunnyvale, CA). The images were processed using Agilent Feature Extraction software (v10.0), and transferred to Agilent Genomic Workbench software for data analysis, CNV visualization, and detection. During the analysis, softwares defaults were not changed and the analysis was based on human genome build hg19/GRCh37. All the assays protocols were performed according to the manufacturer’s instructions (Agilent Inc.).

### Fish

Confirmatory Fluorescence in situ hybridization (FISH) analysis was performed using p-arm and q-arm specific probes for the all chromosomes using standard protocols (Abbott Laboratories, Abbott Parl, IL, USA). In the absence of ready probes, custom FISH probes were designed and used. The experiments were carried out standard protocols.

## Results

### Case 1

The patient is now a 13 year old male who was initially admitted to the hospital for percutaneous valve implantation. He was born with pulmonary atresia with ventricular septal defect (VSD) that was completely repaired with RV to PA conduit in July 2003. He did not suffer any significant cardiac complications, and the follow-up examination reported no cardiac symptoms but the patient had speech delays. Cardiovascular examination showed S1 and S2 were normal with systolic murmur in the left lower sternal border. Echocardiogram revealed normal biventricular systolic function. Right-sided ventricular chambers were mild to moderately dilated but the dilatation has improved post-PPVI. Respiratory and gastrointestinal examinations were insignificant. The patient had speech and developmental delays and brain malformation was suspected. He is mentally retarded. An MRI examination revealed a nonspecific right frontal centrum semiovale hyperintense lesion.

**Fig. 1:**
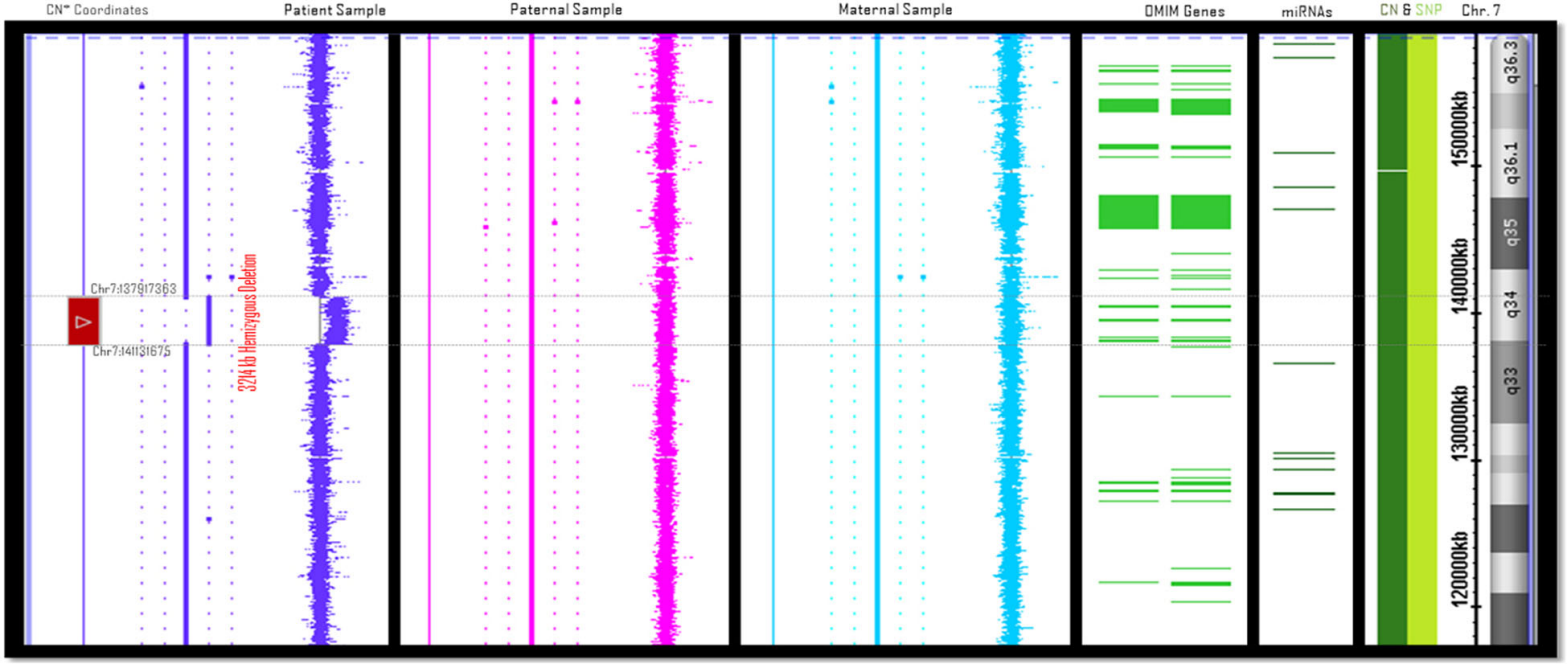
The visual diagram is adopted from Chromosome Analysis Suite (Affymetrix Inc.,). From right to left the diagram presents copy number coordinates, the patient’s probe distribution, paternal and maternal probes distributions, OMIM genes, miRNAs, all SNP and copy number probes in the region, and chromosomal coordinates. The patient has 3214 kb deletion (presented in blue color) while father and mother are normal

Molecular cytogenetic analysis indicated that he has a novel 3.2 Mb deletion extending on 7q33-q34 (Fig. 1). The deletion begins at 137,917,363 bp genomic position and ends at 141,131,675 bp. The deleted region contains 44 genes including 12 uncharacterized genes, 20 pseudogenes, and one miRNA according to Mapviewer Human Annotation Release 107.

### Case 2

The patient is a 13-years old female referred to our center for evaluation of dysmorphic features and congenital heart disease. She was born at term with uneventful prenatal and peripartum period. She was noticed after birth to have hypoplastic right thumb. Her parents, who are not related, have 4 other normal children. When she was initially evaluated in our center at the age of 6 and ½ years, her examination showed a head circumference of 45 cm (4.7 SD below the mean), a weight of 14.6 kg (4 SD below the mean) and a height of 100.5 cm (3 SD below the mean). She had upslanting palpebral fissures, bulbous nose, malformed right ear, retrognathia, low posterior hairline, webbed neck, and widely spaced nipples. The chest and abdominal examination was unremarkable. The cardiovascular examination revealed normal first heart sound, fixed split of second heart sound, and a systolic murmur grade 3/6 over the left upper sternal border. She had normal tone, power and deep tendon reflexes. The musculoskeletal examination revealed right thumb hypoplasia with absent thenar muscles, absent extensor pollicis longus, and thumb extensors. There was significant instability of the metacarpophalangeal joints of the right thumb. Skeletal survey revealed ankylosis between C3, C4 and C5 spine. The thoraacolumbar spine and the long tubular bones of both upper limbs were osteopenic. The right fifth metacarpal bone was short with hypoplasia of the first right metacarpal bone. The epiphysis of the first metacarpal bone was absent bilaterally. There was mild bilateral subluxation of the hip joints. The tibia and fibula were normal bilaterally. Hallux valgus at the interphalangeal joint was seen bilaterally. There was coning of the epiphysis of the second to fourth toes bilaterally. Echocardiogram revealed large secundum atrial septal defect measuring 12 mm with left to right shunt. Ultrasound of abdomen and pelvis indicated that the left kidney was rotated and ectopic lying down into the pelvis.

**Fig. 2:**
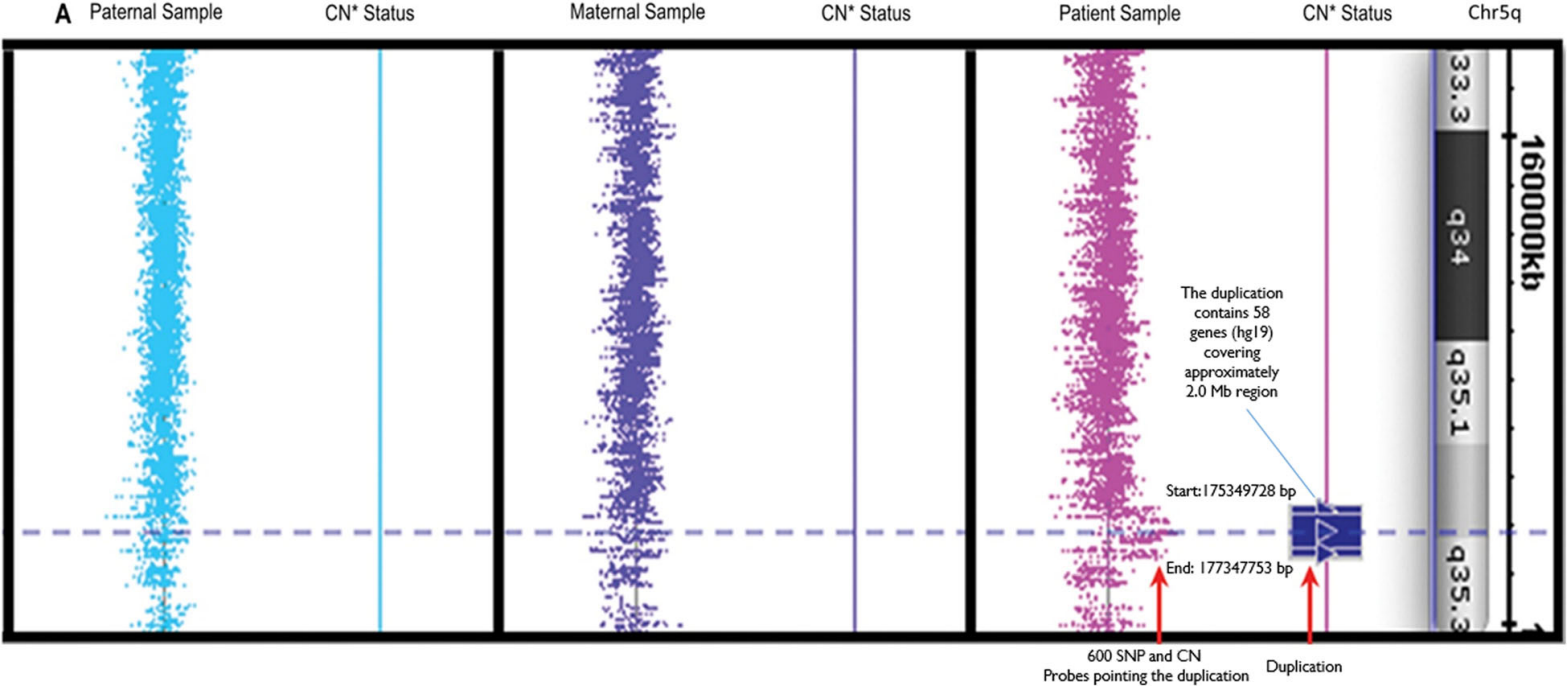
The diagram presents interrogated region, results of the patient’s and parental samples. Copy number status is given next to each tested sample. The patient a duplication comprising more than 50 genes and expanding on approximately 2 Mb region on chromosome 5q35.3. Apparently, parental samples do not carry the gain indicating de novo status of the duplication. The visuals are adopted from Chromosome Analysis Suite (Affymetrix Inc.)

As part of routine diagnostic procedures a high-resolution GTW-banding study was carried on the patient’s sample. No gross abnormality was detected. Then, an aCGH experiment was performed as a further clinical screening and indicated an interstitial duplication pointed by 33 oligonucleotide probes on 5q35.2-q35.3. An interphase FISH using a probe (CTD-2301A4) within the duplicated interval re-confirmed the findings. To better characterize the duplication a high-resolution array (Cytogenetics Whole-Genome 2.7 M) from Affymetrix Inc. (Affymetrix Inc., San Paolo, CA, US) was utilized to further delineate the gain missed during the initial standard microscope based-karyotyping. This particular chip array utilizes 2.7 million markers including 400,000 SNP probes that provide whole-genome coverage with the one of the highest density coverage among the present platforms. Based on Affymetrix’s cytogenetic assay results, the duplication extends from 175,349,728 to 177,347,753 bp (hg19) and comprises 58 genes targeted by more than 600 SNP and copy number (CN) probes covering approximately 2.0 Mb (Fig. 2). In comparison to the previously published duplications this duplication seems novel and does not share breakpoints with the compared cases.

### Case 3

A 5- year-old Saudi male, the first child of non-consanguineous healthy parents, was born at term following in-vitro fertilization (IVF) pregnancy via cesarean section. His birth weight was 3.5 Kg. He was admitted to the neonatal intensive care unit for 1 week because of Jaundice, treated with phototherapy, and was discovered to have congenital heart disease (patent ductus arteriosus [PDA]). Since early infancy, he was noted to have slow psychomotor development, sitting at 10 months and starting to walk independently at 2 years of age. He had significant delay in initiation of language which he developed. At age 1 month, he was admitted to the hospital with febrile illness and treatment as a case of sepsis. When he was 2 years of age, he underwent surgery to place testes (bilateral orchidopexy). There is no previous history of convulsion; however, recently he developed one episode of unprovoked convulsion with semiology of cyanosis and jerky movements of the limbs. Mother had history of two abortions following IVF pregnancy, and there is no family history of epilepsy or neurological problems. Examination at the age of 4 ½ years revealed no dysmorphic features and no neurocutaneuos marks apart from a single hypo pigmented patch at the right forearm. His growth parameters were: weight 18.2 Kg (75th centile), height 110 cm (75th centile), and head circumference 50 cm (50th centile). Vision and hearing were normal. Cardiovascular examination showed apex beat in the fifth intercostal space within the midclavicular line. There were no thrills, no left parasternal heave or palpable P2. Auscultation revealed no murmurs. Neurological examination revealed no gross abnormalities. Laboratory investigations (including complete blood count [CBC], renal function tests, bone profile, liver function tests [LFT], thyroid function tests, and serum lactate and ammonia) were all normal. Brain magnetic resonance imaging (MRI) and magnetic resonance angiography (MRA) were unremarkable. Electroencephalography (EEG) showed normal findings. Recently, the patient had psychometry with IQ score of 57. He was also evaluated by a cardiologist and echocardiogram revealed a small PDA (1.5 mm) with good cardiac functions.

**Fig. 3:**
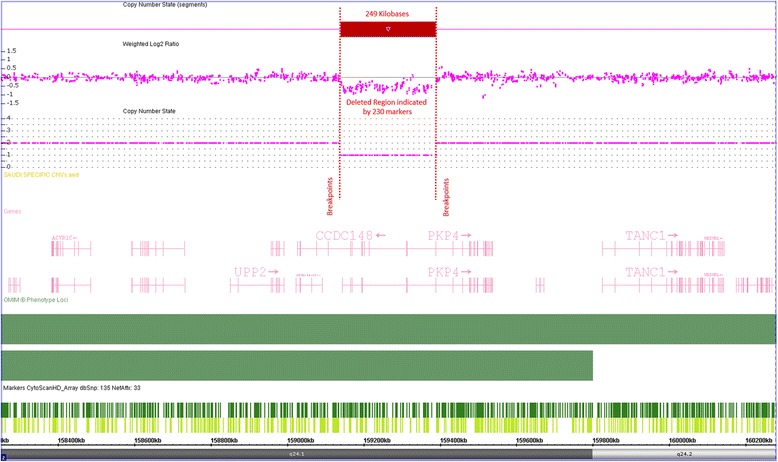
Microarray results are displayed for chromosome 2q23.3–24.3 bands. A deletion is seen on 2q24.1 cytoband expanding over more than 249 kb genomic region

**Fig. 4:**
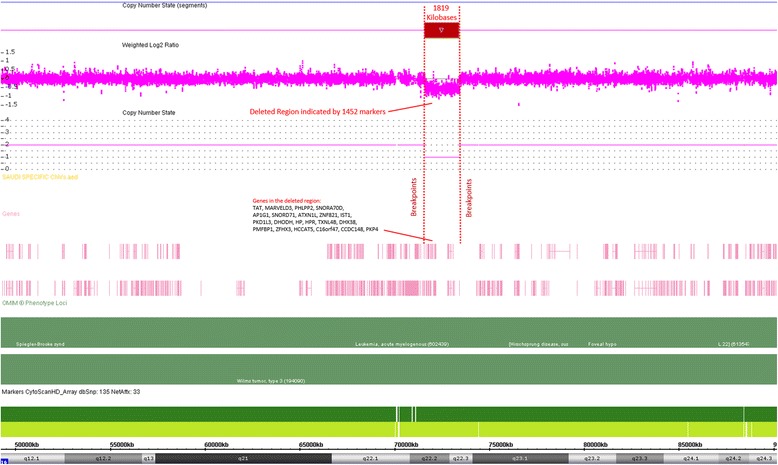
Microarray result displays for chromosome 16. The second deletion on the patient was observed on chromosome 16q22.2–22.3 bands. The deletion is over 1800 kb detected by more than 1450 molecular markers

Whole genome screening of chromosomal aberrations using Affymetrix’s cytogenetic microarrays revealed presence of two large hemizygous deletions at chromosomes 2 (2q24.1 Size: 249 kb) and 16 (16q22.2 Size:1819 kb). Genomic locations of the deletions are chromosome 2, positions: 159,140,953–159,390,141 bp, and chromosome 16 positions: 71,589,375–73,408,685 according hg19 (Figs. 3 and 4). The deleted region in chromosome 2 contains 2 genes and chromosome 16 comprises 18 genes, 5 of which are OMIM-annotated and associated with Tyrosinemia type II (OMIM#: 613,018), Anhaptoglobinemia (OMIM#: 140,100), Hypohaptoglobinemia (OMIM#: 140,100), Prostate cancer susceptibility (OMIM#: 104,155). The patient’s mother was also tested and found negative for above mentioned deletions. Unfortunately, paternal DNA sample was not available for testing and we were unable to recruit the father for further investigation.

### Case 4

This is a 7-year-old boy who was the product of full normal spontaneous vaginal delivery with a birthweight of 3.5 kg. He was well until the age of 8 months when he was noticed to have flexion swinging movements in the hands and wrists that were spontaneous and exaggerated by irritability. There were no other abnormal movements or seizures. He was delayed in attaining milestones with severely impaired cognitive, linguistics and social skills. He was also diagnosed with atrial septal defect. His parents are first-cousins. They have 2 other children who are alive and well. There was no family history of a similar disease. On examination, his head circumference was around 50th percentile, weight was at the 97th percentile, and height was just above the 97th percentile. He had low-set ears and prominent philtrum. His tone, power and reflexes were normal. There were no neurocutaneous manifestations. Skeletal survey, brain MRI and ultrasound of abdomen were normal.

Molecular cytogenetic studies identified a 3.35 Mb interstitial deletion on the long arm of chromosome 3 from 112,146,815 bp to 115,496,750 bp (3q13.2q13.31) (hg19). This region contains over 27 genes, one of which is an annotated OMIM disease gene (*DRD3*). All remaining regions did not show any significant DNA copy number gains or losses.

### Case 5

This is a three-year-old girl who was a product of an in vitro fertilization delivered at 34 weeks of gestation to consanguineous parents with negative family history. She was noticed to have dysmorphic features with low set ears, depressed nasal bridge, and long philtrum. Ophthalmological examination revealed left choroidal coloboma involving the macula and optic disc. Echocardiogram showed large atrial septal defect, large, perimembranous ventricular septal defect, hypoplastic right upper pulmonary vein. She had surgical intervention with ASD and VSD closure and repair of upper pulmonary vein stenosis. She has had global developmental delay and growth failure with all the parameters below the 5th percentile (head circumference − 2.7 SD, weight -6SD, height -4SD). Brain MRI and ultrasound abdomen were normal.

Based on 20 metaphase cells, standard g-banding karyotyping at 425 band resolution indicated an apparently balanced translocation between the q-arm of chromosome 10 and the q arm of chromosome 12 (46,XX,t(10;12)(q22;q22)) was found in all the cells; however, loss of chromosomal material cannot be ruled out based on the low band resolution seen. Although this translocation could be de-novo most likely one of the parents is a carrier for the translocation. Follow-up aCGH study indicated presence of two deletions; one on chromosome 12 (12p12.1p11.21; 25,320,816–31,285,151; ~5.96 Mb) and the other on the chromosome 16 (16p11.2; 29,567,295–30,321,320; ~754 Kb), (hg19). The deletion on chromosome 16 is paternally inherited. The larger deletion (chromosome 12) observed in this patient is not present in either parent and therefore appears to be a de novo event.

### Case 6

This is a 4-year-old female who was the product of full normal spontaneous vaginal delivery with a birthweight of 1.45. She stayed in the NICU for 2 months and was diagnosed with perimembranous ventricular septal defect. She was noticed to have global developmental delay and poor growth. There was no history of seizures. The parents are not consanguineous. There is no family history of a similar problem. Her physical examination was notable for microcephaly (7 SD below the mean). Her weight was -5SD and height was -3SD. She had low anterior hair line, squint, broad nasal bridge, short philtrum, and micrognathia. The muscle tone was mildly increased in the upper and lower extremities. Ultrasound of abdomen showed mild right upper pole caliectasis.

**Fig. 5:**
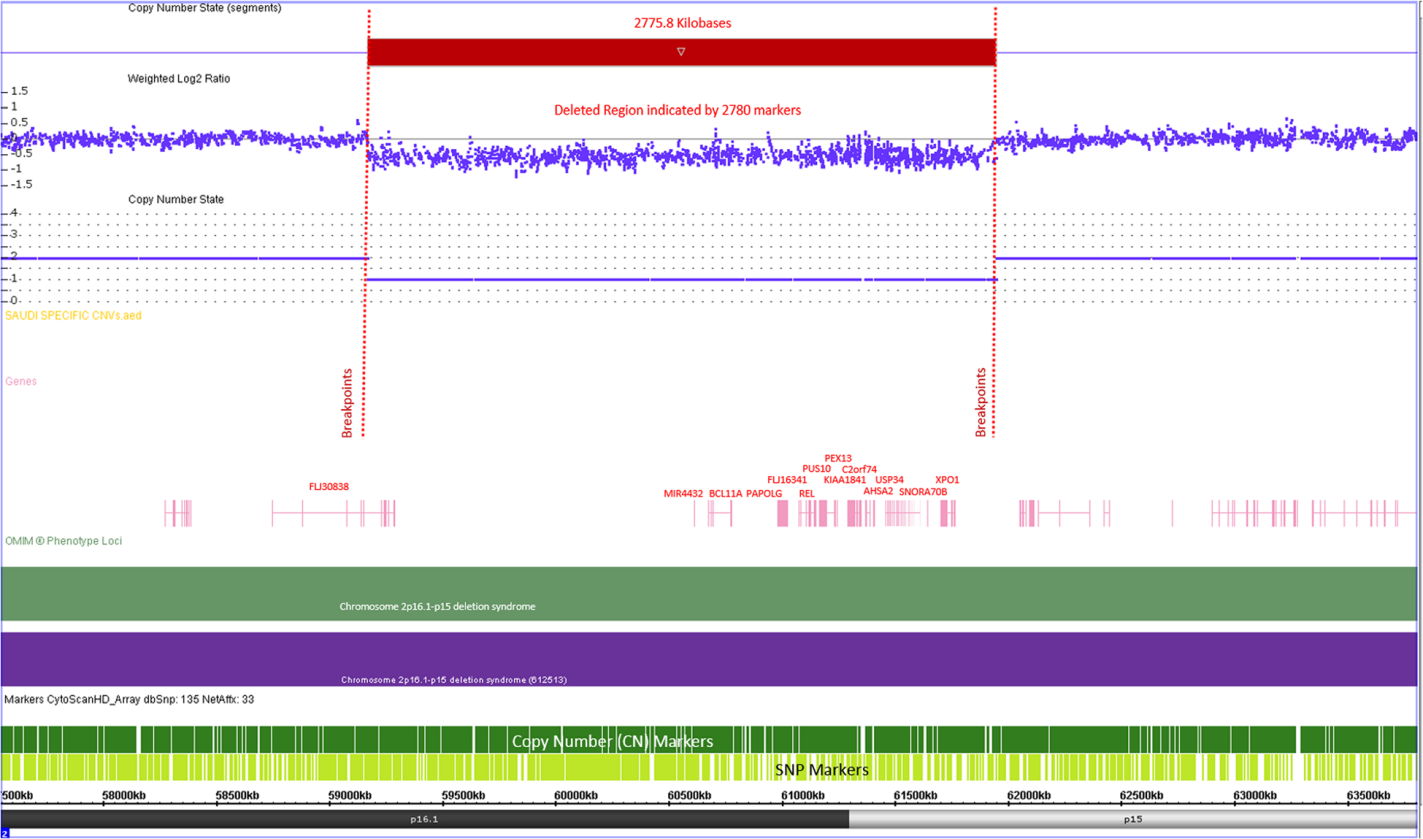
A deletion on chromosome 2p16.1-p15 is presented in the diagram. The deletion comprises several genes and ~2776 kb genomic region, and detected by 2780 SNP and CN probes

Molecular cytogenetic analysis revealed presence of 2.78 Mb interstitial deletion in the short arm of chromosome 2 (extending between 59,170,950 bp and 61, 946, 784 bp over chromosome 2p16.1 deletion syndrome region) (Fig. 5). Although the region is large in size, it is relatively gene-poor region with a total of only 27 genes, six of which are OMIM-annotated. Among these *PEX13* is known to associate with Zellweger syndrome.

## Discussion

### Case 1

Deletions in the 7q33-q34 region are rarely reported in the scientific literature. Reported deletions in this region are mostly associated with developmental delay, intellectual disability, microcephaly, and significant morphological and developmental phenotypes. The deleted region in this case contains 50 genes including the *BRAF*; the mutation of which is known to be associated with cardiofaciocutaneous (CFC) syndrome [23], a disease characterized by heart defects, mental retardation and a distinctive facial appearance. *BRAF* encodes for the BRAF protein, which is involved in the MAP kinase/ERK signalling pathway; an important pathway that implicates various cell processes including growth, differentiation, proliferation, senescence and apoptosis [24]. Mutations in *BRAF* disrupt the regulation of MAP kinase/ERK pathway and can lead to a range of complications including various types of cancers as well as developmental disorders such as Noonan syndrome (NS), Costello syndrome, LEOPARD syndrome, and Cardiofaciocutaneous syndrome (CFC). Interestingly, only one of the previously described cases shared a deletion in the genomic region constituting the *BRAF* gene [25]. This makes it a likely candidate to explain the clinical features in these cases.

### Case 2

Chromosome 5q35.2-q35.3 deletions are well-known mainly due to Sotos syndrome. Altogether, these genomic alterations reach to a significant number [26–28]. Compared to deletions [27, 29–33] duplications in the region are rare and not well-characterized [34–37]. Moreover, there is no well-established genotype-phenotype correlation for these gains currently since they are in variable sizes and lack precise breakpoints. Interestingly among these cases only singleton have been reported to have Sotos syndrome-like symptom [38]. The rest of cases have different phenotypic findings mostly in the form of developmental delay and short stature. Among these cases, two duplications exceed nearly twice the size of the rest of the gains located on the 5q35.2-q35.3 region [34, 38]. In the present study we describe a patient with a duplication leading to congenital heart disease, cervical ankylosis, and thumb hypoplasia in addition to microcephaly, short stature, and various dysmorphic features. Intriguingly, among the duplication carrying patients, beside our case, there are only three patients who have heart defects [38, 39].

In their study Jamsheer et al. [38] pointed out likely involvement of *MSX2* in radial agenesis as well as complex heart defect, and *FGFR4* as causative factor of limb formation. Although *FGFR4* is shared by both gains (ours and that of Jamsheer et al. [38]), *MSX2* is located outside the boundaries of our duplication. Deletions of both genes, *NSD1* and *FGFR4,* were previously reported with congenital heart anomalies [40]. However, interestingly, *FGFR4* is not a shared gene between all four cases having heart defects. In other words it is not in the shared region of the patients reported in Rosenfeld et al.’s study [39]. Hence, involvement of this gene in the reported heart defects is less likely. Relatedly Rosenfeld et al. raised the likely contribution of another candidate gene *PDLIM7* which is shared among all the cases with the heart defect including ours according to recent human assembly (hg38, 39]. *PDLIM7* is a scaffold protein that regulates *Tbx5* which has critical roles in heart and limb development. Moreover, suppressed expression of *Pdlim7*in zebrafish led to the development of heart abnormalities in the animals.

### Case 3

The deletion is large (1.8 Mb) and comprises 18 genes (*TAT*, *MARVELD3*, *PHLPP2*, *SNORA70D*, *AP1G1*, *SNORD71*, *ATXN1L*, *ZNF821*, *IST1*, *PKD1L3*, *DHODH*, *HP*, *HPR*, *TXNL4B*, *DHX38*, *PMFBP1*, *ZFHX3*, *HCCAT5*, *C16orf47*) located in 16q22.1-q24 cytogenetic region. Such deletions are frequently seen among breast cancer patients [41]. Moreover, two different cases having cytogenetic abnormalities in the long arm of chromosome 16, were previously described with ventricular septal defect [42, 43]. However, these abnormalities are not overlapping with 16q22.2 cytogenetic band.

The partial deletion of 16q is implicated in the rare 16q22 deletion syndrome (OMIM #614541) characterized by failure to thrive, growth retardation, dysmorphic facial features, and hypotonia. *DHODH* is one of the implicated genes that encodes for dihydroorotate dehydrogenase which catalyzes the oxidation of dihydroorotate to orotate, thereby facilitating the biosynthesis of pyrimidine blocks. Moreover, the mutations in *DHODH* lead to Miller syndrome, also known as postaxial acrofacial dysostosis [44]. Interestingly, it was also found that DHODH is involved in the transcriptional elongation of *BRAF* [45] that is a well-known oncogene, a member of the Raf kinase family, and an important molecule for RAS/MAPK signaling pathway. Mutations in this gene cause different hereditary disorders such as cardiofaciocutaneous syndrome, multiple lentigines syndrome, and Noonan syndrome as well as the development of birth defects.

Small deletions in the 2q24.1q24.2 region are quite rare [46]. A female patient was screened with SNP arrays and found to carry de novo deletion of 2q24.1q24.2 region. The patient had mental retardation and generalized hypotonia but lacking any cardiovascular problem [46]. The deleted region on chromosome 2 in our patient harbors two genes: *CCDC148* and *PKP4*. *PKP4* has been speculated to be a modifier gene for *LMNA* in which a splicing mutation caused sudden death, ventricular arrhythmia, cardiomyopathy, and heart failure in a 63-year-old male with a family history of individuals (>10) with similar problems [47].

It is also noteworthy to mention that paternal DNA sample was not available for cytogenetic testing. Hence, we were unable to confirm the de novo status of the deletions in our patient.

### Case 4

Our molecular cytogenetic studies identified an interstitial microdeletion on 3q13.2q13.31 cytobands. Such deletions are rare [48] and only few cases have been reported by now. There are more cases of larger deletions in the region (3q11q23) with a range of various phenotypic features such as developmental delay, facial dysmorphisms, and musculoskeletal abnormalities [49–54]. A recent study summarized most of these cases excluding few recently reported patients [55–57]. The study narrowed down all the deletions to a shared region that harbors expectedly significantly lesser genes than those of the larger region from the previous 3q deletion studies [58]. In this study the smallest deletion was nearly 0.6 Megabases of size and located on 3q13.31. This region contains over 27 genes, one of which is an annotated OMIM disease gene (*DRD3*). Genotype-phenotype comparison of more than 20 patients shared 3q13.31 deletion and all shared some common phenotypic features developmental delay, muscular hypotonia, a high arched palate, and recognizable facial features including short philtrum and protruding lips. Heart related abnormalities were not among the listed characteristics. The authors speculated that developmental delay seen in these patients is related to *DRD3* and *ZBTB20*. Intriguingly none of these cases of 3q13.31 deletion have heart related abnormality. Moreover, considering that the parents are closely related, the phenotype is likely to originate from the consanginuity. However, this needs further investigations.

### Case 5

Chromosome 12p (12p12.1p11.21) and chromosome 16p (16p11.2) deletions are not commonly co-occurring. There are reports for deletions for 12p and 16 p regions [59, 60]. There is only a single report of an interesting patient, who harbors two-hits, maternally inherited 16p13.11-p12.3 duplication and a de novo 12p12.1 deletion [61] whereas our patient has a paternally inherited 16p11.2 deletion and a de novo 12p12.1p11.21 deletion. However, considering the gain type and affected cytogenetic bands our patient is unique and will add to literature of two-hits patients. Deletions of 16p11.2 have been associated with the highly variable phenotype ranging from intellectual disability autism and congenital abnormalities to mildly affected or unaffected cases [62]. In such cases a child may be on one and of the conical spectrum. A recent comprehensive study revisits deletions and duplications in this region in 246 patients. The interesting difference between carriers of the 16p11.2 deletions and duplications is the frequent encounter of macrocephaly among the deletion cases [63]. However, the larger deletion (Chromosome 12) observed in this patient is not present in either parents and therefore appears to be a de novo event as such it is likely to be a significant contributor to the patient’s phenotype. Chromosome 12p12.1 deletions harboring SOX5 have been previously reported [64–66]. Among these few patients were reported to have heart related problems such as ventricular septal defect, slight arrhythmia, secundumatrial septal defect, and atrioventricular canal [66]. Phenotypic consequences of these patients were linked to SOX5 haploinsufficiency.

### Case 6

2p16.1p15 deletion harbors 27 genes including *PEX13*. While compound heterozygous and homozygous mutations in *PEX13* are associated with Zellweger syndrome (Type PDB11A (OMIM#614883). Haploinsufficiency of this gene due to a heterozygous deletion has not been reported to be a cause of the disease. A *PEX13* sequence based mutation on the non-deleted chromosome could conceivably give rise to a clinical phenotype that differs from Zellweger syndrome and sequencing of this gene is considered. However, the plasma very long chain fatty acids assay was normal in the patient; hence, the sequencing of *PEX13* was not performed. Until recently, microdeletions of 2p15–16.1 were identified in 15 patients with a recognizable syndrome of dysmorphic features, microcephaly and intellectual disability [67] in addition to the patients deposited to the public databases such as DECIPHER and ISCA. Among the cases, no patient has a report of heart related defect. Hence, the relationship of this deletion to our patient’s phenotype needs further delineation. Moreover, parental studies would be useful in order to determine whether this alteration represents a familial variant or a de novo change. De novo changes are more likely to be clinically significant.

## Conclusions

In conclusion, we present the first chromosomal imbalances associated with congenital heart abnormalities among Saudi patients. Such information, combined with further delineation of similar cases and relatedly collection of Saudi Specific CNVs, will allow better understanding of the pathobiology as well as management of the CHD patients in Saudi Arabia.
